# Comparison of the Metabolites in Fingered Citron Fruit (*Citrus medica* L. var. *sarcodactylis* Swingle) and Chayote (*Sechium edule*) Based on UPLC‐Q‐Orbitrap MS/MS

**DOI:** 10.1002/fsn3.72115

**Published:** 2026-07-16

**Authors:** Bin Li, Ruiyi Fan

**Affiliations:** ^1^ College of Food and Biology Jingchu University of Technology Jingmen Hubei China; ^2^ Hubei Engineering Research Center for Specialty Flowers Biological Breeding Jingchu University of Technology Jingmen Hubei China

**Keywords:** chayote, *Citrus medica*, food authentication, regional quality discrimination, untargeted metabolomics

## Abstract

Fingered citron ( *Citrus medica* L. var. *sarcodactylis* Swingle) is a medicinal and edible citrus fruit cultivated in several major production regions of China. However, comprehensive information on its regional metabolite variation and its chemical differentiation from chayote ( *Sechium edule* ), a botanically unrelated commodity that may be confused with fingered citron because of partially overlapping Chinese vernacular names, remains limited. In this study, untargeted UPLC‐Q‐Orbitrap MS/MS was used to profile fingered citron samples from Zhejiang (CF1), Sichuan (CF2), Guangdong/Guangxi (CF3), and Yunnan (CF4), together with chayote samples used as a targeted authentication comparator (CFM). A total of 380 metabolites were putatively annotated, including 47 secondary metabolites comprising 23 flavonoids, 8 terpenoids, 8 phenols, 3 coumarins, 3 alkaloids, and 2 steroids. Multivariate analyses and hierarchical clustering identified 65 differential metabolites, including 19 secondary metabolites, that clearly separated the five sample groups within the present dataset. The very high cross‐validated *Q* ^2^ values should nevertheless be interpreted cautiously because of the large taxonomic and metabolic distance between *C. medica* and *S. edule* and the limited sample size. The interspecific separation was therefore interpreted as an expected chemotaxonomic difference with practical authentication value, rather than as a comparison between biologically equivalent taxa. Phenylalanine metabolism and flavone and flavonol biosynthesis showed the strongest nominal enrichment, although neither remained significant after FDR correction. These results provide a metabolite reference for regional quality discrimination of fingered citron and identify candidate chemical features for its targeted authentication against chayote. Further validation using independent samples, additional production years, and other potential comparator commodities is required before routine application.

## Graphical abstract

Untargeted UPLC‐Q‐Orbitrap MS/MS metabolomics was used to profile fingered citron ( *Citrus medica* ) from four Chinese production regions (CF1–CF4) and chayote ( *Sechium edule* , CFM). Of 380 putatively annotated metabolites, 65 differential metabolites distinguished the five groups, clearly separating chayote from fingered citron and highlighting regional chemical variation among fingered citron cultivars.

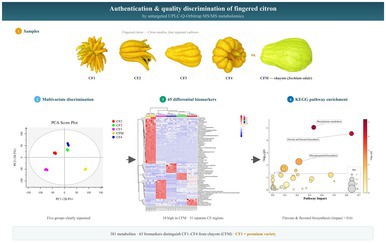

## 1 Introduction

Fingered citron (CF) ( *Citrus medica* L. var. *sarcodactylis* Swingle) also known as “Foshou” in China, is one member of the three ancestral species of *Citrus* genus (Mahdi, Al‐Ansi, Al‐Maqtari, et al. 2019). About 300 years ago, CF was merely cultivated as ornamental plant (Sottile et al. 2019). Nowadays, it is also popular as an edible medicinal fruit with names as “Buddha hand citron,” “Longevity orange,” and “Five finger orange” (Peng et al. 2009), and its name is usually misapplied as bergamot ( *Citrus bergamia* Risso) (Peng, Luo, et al. 2019; Peng, Yang, et al. 2019), nevertheless, their appearance is quite different. And they are two different kinds of fruits in *Citrus* genus (Donna et al. 2011). CF fruit has been utilized in some ingredients of Traditional Chinese Medicine (TCM) for the treatment of various chronic diseases including hypertension, respiratory tract infections, and type 2 diabetes mellitus (Peng et al. 2009). The bioactivities of CF are attributed to its bioactive compounds such as terpenes, flavonoids, coumarins and polysaccharides (Wu et al. 2013). The volatile compounds and essential oils of CF have drawn great attention of the researchers (Gao et al. 2020; Luo et al. 2019). Chemical composition and bioactivities of essential oils from CF have been studied in detail from all respects (Mahdi, Al‐Ansi, Ahmed, et al. 2019; Li et al. 2019; Xu et al. 2019). Besides, isolation, structural characterization and immunoregulatory activities of polysaccharides from CF have also been examined (Peng, Luo, et al. 2019; Peng, Yang, et al. 2019). However, the investigation of other chemical components such as flavonoids and coumarins which are also highly available in CF is limited (Chen et al. 2020). Recent comparative metabolomic and quantitative phytochemical studies have begun to broaden this characterization, expanding the chemical map of CF and linking specific flavonoids and coumarins to growth‐period‐dependent antioxidant capacity (El Sayed et al. 2024; Tan et al. 2025). Hence, there is a need to conduct comprehensive investigation for the chemical constituents of CF fruits. However, a comparative metabolomic investigation covering the major Chinese production regions of CF remains lacking. In addition, the potential of metabolomic profiling to distinguish CF from chayote ( *Sechium edule* ) has not been systematically evaluated. Although *S. edule* is taxonomically distant from *C. medica* , it is known in Chinese as “Foshougua,” whereas CF is commonly known as “Foshou.” This partially overlapping vernacular terminology, together with potential superficial confusion during commodity identification, makes *S. edule* a practically relevant comparator for a targeted authentication analysis. The purpose of such a comparison is not to treat the two species as biologically equivalent, but to evaluate whether their expected chemical differences can provide reproducible features for commodity discrimination.

In China, CF fruits are cultivated in different regions, and each of them develops its own regionally distinct types with different fruit morphologies. To date, there are four main production regions of CF in China. Cultivated in the provinces of Guangdong and Guangxi, CF is called “Guang” CF, and that grown in Sichuan province is named “Chuan” CF, and in the provinces of Yunnan, they are called “Yun” CF, respectively. In addition, “Jin” CF is named according to the main production city of Jinhua in Zhejiang province.

In the present study, CF samples from Zhejiang, Sichuan, Guangdong/Guangxi, and Yunnan were designated CF1, CF2, CF3, and CF4, respectively. Chayote ( *S. edule* , CFM) was selected a priori as the sole non‐*Citrus* comparator because it represents the specific commodity‐identification issue addressed in this study, rather than because it is taxonomically or biologically equivalent to CF. Untargeted UPLC‐Q‐Orbitrap MS/MS metabolomics, together with PCA, PLS‐DA, OPLS‐DA, hierarchical clustering, and pathway analysis, was used to characterize the metabolite profiles of these samples. The objectives were to (i) assess regional chemical variation among the four CF groups, (ii) identify candidate metabolites associated with regional quality discrimination, and (iii) evaluate whether metabolite fingerprints could support targeted authentication of CF against chayote. The conclusions concerning authentication are restricted to the sample groups examined in this study and require further external validation.

## 2 Materials and Methods

### 2.1 Plant Materials and Chemical Reagents

CF ( *C. medica* ) fruits were purchased from the local markets at different regions of China. Six biological replicates were analyzed for each sample group (CF1–CF4 and CFM). The fruits were chopped into small blocks, then they were frozen in liquid nitrogen immediately, and stored in −80°C for further analysis. The botanical identity of *C. medica* var. sarcodactylis and *S. edule* samples was confirmed by the corresponding author (R.F.) based on diagnostic morphological characteristics of the fruits; no voucher specimens were deposited because the samples were purchased as market commodities.

Acetonitrile and methanol with purity higher than 99.0% were purchased from Thermo Fisher Scientific Inc. (Shanghai, China). 2‐chlorophenylalanine was obtained from Aladdin Reagent Co. Ltd., China. Formic acid of LC–MS grade was obtained from TCI Shanghai. Ammonium formate was bought from Sigma‐Aldrich Inc. (St. Louis, MO, USA).

### 2.2 Extraction Procedure

Briefly, accurately weigh 100 mg (±1%) of tested sample, and add 1.2 mL 2‐chlorophenylalanine (4 ppm) methanol (precooled at −20°C), vortex for 30 s. Then 100 mg glass beads were added and the mixtures were ground at 25 Hz for 60 s in a tissue grinding machine (TissueLysis II). Subsequently, the samples were ultrasonicated at room temperature for 15 min. Afterwards, centrifugation was conducted at 25°C for 10 min at 1750 g, and the supernatant was filtered through a 0.22 μm membrane to obtain the prepared samples for LC–MS. The extract of 20 μL from each sample was mixed all together to make quality control (QC) samples.

### 2.3 UPLC‐Q‐Orbitrap MS/MS and Data Analysis

Chromatographic separation was performed on a Vanquish UHPLC system (Thermo Fisher Scientific) coupled to a Q Exactive HF‐X mass spectrometer (Thermo Fisher Scientific) operating in full MS/data‐dependent MS2 (ddMS2) mode. Separation was achieved on a Waters ACQUITY UPLC HSS T3 column (100 mm × 2.1 mm, 1.8 μm) maintained at 40°C. Mobile phase A consisted of 0.1% (v/v) formic acid in water containing 5 mmol/L ammonium formate, and mobile phase B was 0.1% formic acid in acetonitrile. The gradient elution programme was as follows: 0–2 min, 5% B; 2–12 min, 5%–90% B; 12–14 min, 90% B; 14–14.1 min, 90%–5% B; 14.1–17 min, 5% B (re‐equilibration). The flow rate was 0.3 mL/min and the injection volume was 2 μL. Mass spectrometry parameters were set as follows: spray voltage, +3.5/−2.8 kV (positive/negative mode); capillary temperature, 320°C; sheath gas flow rate, 45 arb; auxiliary gas, 10 arb; full MS scan range, *m*/*z* 100–1200; MS2 resolution, 17,500; normalized collision energy (NCE), 20, 40, and 60 eV (stepped). Peak identification, filtration, and alignment were conducted as described previously (Fan et al. 2020). Identification, filtration and alignment of peaks were conducted via XCMS (www.bioconductor.org) using R language (Smith et al. 2006). Data array of mass to charge ratio (m/z), retention time and peak intensity was subsequently obtained, and 13,947 precursor molecules in positive ion mode and 5435 precursors in negative ion mode were acquired for further analysis. Annotation of each metabolite was carried out according to their exact molecular weights and fragmentation pattern compared with the compounds in public spectral libraries, namely mzCloud (https://www.mzcloud.org), the Human Metabolome Database (HMDB), Metlin, MassBank and Lipid Maps, together with an in‐house spectral database built by Suzhou Bionovogene Co. Ltd. (Fan et al. 2020). All metabolite annotations reported in this study are based on MS^2^ spectral‐library matching without confirmation using authentic chemical standards. According to the Metabolomics Standards Initiative (MSI) guidelines (Sumner et al. 2007), these annotations correspond to confidence Level 2 (putatively annotated compounds).

### 2.4 Multivariate Statistical Analysis

The output data array from XCMS was processed in Microsoft Excel (Microsoft, Redmond, WA, USA) and all the data were mean‐centered and unit variance (UV)‐scaled for multivariate statistical analysis including principal component analysis (PCA), partial least squares‐discriminant analysis (PLS‐DA), and orthogonal partial least squares discriminant analysis (OPLS‐DA). All data were mean‐centered and UV‐scaled before multivariate statistical analysis. Sevenfold cross‐validation was used to assess the supervised models, and the PLS‐DA model was additionally evaluated using 100 random permutation tests as an internal diagnostic of model stability. Then, the normalized data was further processed by Simca‐P software version 13.0 (Umetrics AB, Umea, Sweden, www.umetrics.com/simca) and *ropls* package (Thévenot et al. 2015). Variables with a VIP > 1 and *p* < 0.05 were considered as significantly different.

### 2.5 Hierarchical Clustering and KEGG Enrichment Analysis

The differential metabolites were used to make the agglomerate hierarchical clustering analysis (HCA). The data arrays were processed by the program of pheatmap in the *ropls* package.

MetPA (MetaboAnalyst 5.0, www.metaboanalyst.ca) was applied to conduct the pathway analysis based on the KEGG annotation (https://www.kegg.jp/) through integrating pathway enrichment analysis and pathway topology analysis. The analysis result was visualized in a bubble plot. The higher the Impact and the lower the *p* value, the more significant the pathway. Raw pathway‐enrichment *p* values were adjusted for multiple testing using the Benjamini–Hochberg false discovery rate (FDR) procedure implemented in MetaboAnalyst 5.0, and an FDR‐adjusted *p* < 0.05 was considered statistically significant.

## 3 Results and Discussion

### 3.1 Phytochemical Profiles

Before test of the samples, QC and quality assurance (QA) were carried out to make sure the reliability of methodology and data as shown in Figure S1. Theoretically, QC samples are all the same, but there will be systematic errors in the sample extraction, detection and analysis process, resulting in differences between QC samples. The smaller the difference, the higher the stability of the method, the better the data quality, which is reflected in PCA analysis as illustrated in Figure S1A indicating that the data are reliable (Dunn et al. 2011). In order to discover biomarkers, the relative standard deviation (RSD) of potential characteristic peaks in QC samples should not exceed 30% or the relevant characteristic peaks should be deleted (Dunn et al. 2011) which is the QA. In Figure S1B, the proportion of characteristic peaks with RSD < 30% is higher than 75% which further validate the reliability of the obtained data (Want et al. 2010).

**FIGURE 1:**
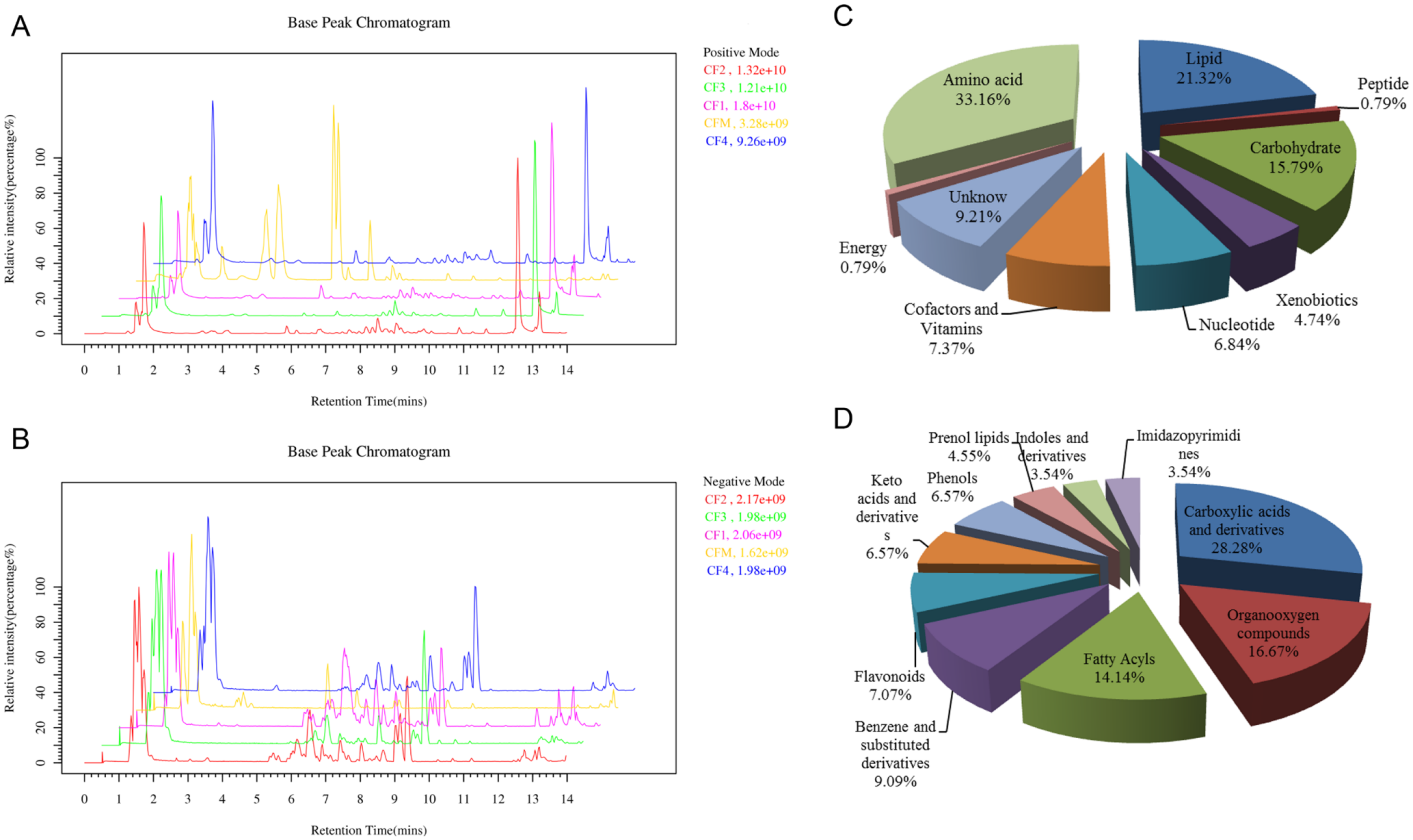
Base peak ion (BPI) chromatograms of four regional fingered citron groups (CF1–CF4) and chayote (CFM) in positive (A) and negative (B) modes, and classification of the metabolites detected into major functional classes (C) and top 10 chemical classes (D).

Figure 1A,B show the typical base peak chromatograms of four regional CF groups and chayote (CFM) in positive and negative ion modes, respectively. After processing the raw data using ProteoWizard Data Analysis software (v3.0.8789) and XCMS, 13,947 and 5435 precursor features were detected in positive‐ and negative‐ion modes, respectively. A total of 380 metabolites, including both primary and secondary metabolites, were putatively annotated across the five sample groups (Table S1), as summarized in Figure 1C. Figure 1D shows the ten most represented chemical classes among the annotated metabolites. It is worth noting that flavonoids and phenols accounted for 3.68% and 3.42% of all annotated metabolites, respectively. These two types of compounds were proven to be bioactive with multiple functions in terms of protecting plants from stress and providing additional health benefits for human beings (Dixon et al. 2002).

To further examine the flavonoids and phenols in CF fruits, all the secondary metabolites were selected and shown in Table 1. A total of 47 secondary metabolites including 23 flavonoids, 8 terpenoids, 8 phenols, 3 coumarins, 3 alkaloids, and 2 steroids were putatively annotated by UPLC‐Q‐Orbitrap MS/MS. In order to compare the contents of secondary metabolites in different regional groups, a rank for the relative amount of individual compounds was performed and the results were presented in Table 1. CF2 from Sichuan and CF1 from Zhejiang showed the two highest cumulative relative‐abundance rankings for the secondary metabolites listed in Table 1. In contrast, CFM showed the lowest overall relative abundance of these secondary metabolites, whereas CF3 and CF4 displayed intermediate profiles.

**TABLE 1:** Main secondary metabolites and their relative‐abundance rankings in four regional fingered citron groups (CF1–CF4) and chayote (CFM).

No.	Name	Classification	CF1	CF2	CF3	CF4	CFM
1	Hesperetin	Flavanone	3	5	2	4	1
2	Neohesperidin	Flavanone 7‐*O*‐neohesperidose	3	5	2	4	1
3	Chrysin*	Flavone	5	4	1	2	3
4	Luteolin*	Flavone	5	3	4	2	1
5	Rhoifolin	Flavone 7‐*O*‐neohesperidose	4	5	3	2	1
6	Scolymoside	Flavone 7‐O‐neohesperidose	5	3	1	4	2
7	Isovitexin*	Flavone 6‐*C*‐glucoside	2	5	1	3	4
8	Apiin	Flavone diglycoside	5	4	3	2	1
9	Quercetin*	Flavonol	5	4	3	1	2
10	Kaempferol	Flavonol	4	1	3	2	5
11	Isoquercitrin*	Flavonol 3‐*O*‐glucoside	5	4	2	1	3
12	Rutin	Flavonol 3‐*O*‐rutinoside	5	4	3	2	1
13	Kaempferide	*O*‐methylated flavonol	3	5	2	4	1
14	3‐*O*‐methylquercetin*	*O*‐methylated flavonol	5	4	2	3	1
15	Pelargonin*	Anthocyanin	5	4	3	2	1
16	Leucopelargonidin	Anthocyanin	5	4	3	2	1
17	Peonidin‐3‐glucoside*	Anthocyanin	3	5	2	4	1
18	Malvidin 3‐glucoside	Anthocyanin	4	2	5	1	3
19	Cyanidin 3‐glucoside	Anthocyanin	3	4	1	2	5
20	Cyanidin 3‐*O*‐rutinoside 5‐*O*‐beta‐d‐glucoside*	Anthocyanin	5	3	1	2	4
21	Cyanidin 3‐*O*‐(2‐*O*‐beta‐D‐glucuronosyl)‐beta‐D‐glucoside	Anthocyanin	4	5	3	1	2
22	Delphinidin 3‐*O*‐beta‐D‐glucoside 5‐*O*‐(6‐coumaroyl‐beta‐d‐glucoside)*	Anthocyanin	5	3	1	2	4
23	(−)‐Epigallocatechin*	Flavan‐3‐ol	2	3	5	4	1
24	Pulegone	Monoterpene	2	5	4	3	1
25	d‐Limonene	Monoterpene	5	3	2	4	1
26	(+)‐α‐Pinene*	Monoterpene	3	5	4	2	1
27	(1S,4R)‐1‐Hydroxy‐2‐oxolimonene	Monoterpene derivative	3	5	2	4	1
28	Limonene‐1,2‐diol*	Monoterpene alcohol	2	4	3	5	1
29	2‐Trans,6‐trans‐farnesal*	Sesquiterpene alcohol	3	5	4	2	1
30	(1R,4R)‐Dihydrocarvone	Ketone	4	2	1	3	5
31	Eucalyptol	Monoterpene ether	2	4	5	1	3
32	Sinapyl alcohol	Phenol	5	4	3	2	1
33	Perillyl alcohol*	Phenol	3	5	2	4	1
34	Coniferyl alcohol*	Phenol	5	3	4	2	1
35	Chavicol	Phenylpropene	1	4	3	2	5
36	Eugenol*	Allylbenzene	4	5	3	2	1
37	Isochavicol	Phenol	1	4	3	2	5
38	Methyleugenol	Phenylpropene	4	2	5	3	1
39	Estragole	Phenylpropene	5	3	4	2	1
40	Coumarin	Coumarin	3	2	4	5	1
41	Scopoletin	Coumarin	4	5	3	2	1
42	Umbelliferone*	Coumarin	5	4	3	2	1
43	*N*‐methylserotonin*	Alkaloid	5	4	3	2	1
44	*N*‐acetylserotonin	Alkaloid	2	5	1	3	4
45	Deoxyloganin	Alkaloid	5	3	4	2	1
46	Campestanol	Steroid	2	3	1	4	5
47	β‐Sitosterol	Steroid	2	5	4	1	3
	Total		175	183	131	120	96

* Nineteen secondary metabolites classified as differential metabolites using VIP > 1 and *p* < 0.05.

In various different types of secondary metabolites, flavonoids cover almost half of the total secondary metabolites which indicated that CF fruits are abundant of flavonoids including flavanones, flavones, flavonols and anthocyanins. Some of the flavonoids were also reported to be contained in other *Citrus* family such as hesperetin, rhoifolin, neohesperidin, luteolin, quercetin, rutin (Soares et al. 2015; Zhang et al. 2012; Olas 2021; Johnson et al. 2011; Fan et al. 2019). These citrus flavonoids exert good physiological activities such as antiproliferative, antitumor (Ferreira de Oliveira et al. 2020), antidiabetic (Rao et al. 2011), neuroprotective (Brinza et al. 2020), prebiotic (Lu et al. 2020) activities. Some of the secondary metabolites such as 3‐*O*‐methylquercetin, pelargonin, leucopelargonidin, peonidin‐3‐glucoside, malvidin 3‐glucoside (1S,4R)‐1‐hydroxy‐2‐oxolimonene, sinapyl alcohol, isochavicol, N‐acetylserotonin, deoxyloganin, campestanol, and β‐sitosterol were tentatively annotated in CF for the first time, to the best of our knowledge. Thus, this untargeted metabolomic analysis expands the available chemical information on CF and provides a basis for its further nutritional and medicinal evaluation. It should be noted that all metabolite annotations were based on MS^2^ spectral‐library matching without confirmation using authentic chemical standards, corresponding to MSI confidence Level 2; therefore, the structural assignments of these tentatively annotated compounds should be interpreted with appropriate caution.

### 3.2 Multivariate Analysis of Annotated Metabolites

**FIGURE 2:**
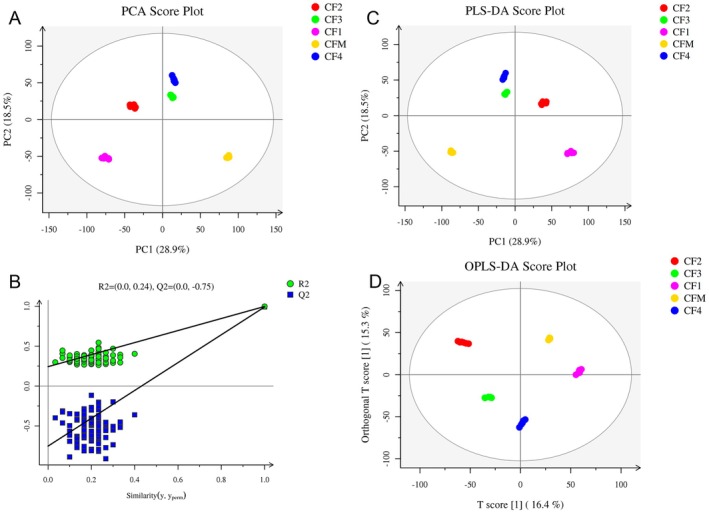
(A) Principal component analysis (PCA), (B) permutations plot of the PLS‐DA model, (C) partial least squares‐discriminant analysis (PLS‐DA), and (D) orthogonal projections to latent structures discriminant analysis (OPLS‐DA) of four regional fingered citron groups (CF1–CF4) and chayote (CFM) for the CF1 versus CF2 versus CF3 versus CF4 versus CFM in positive ion mode.

PCA was first conducted to evaluate the overall variation in the annotated metabolite profiles without using prior group information. As shown in Figure 2A, CF2, CF3, and CF4 clustered relatively closely, whereas CF1 was more clearly separated from these three regional groups. CFM was distinctly separated from all four CF groups. Given that *C. medica* and *S. edule* belong to taxonomically distant families and orders, this interspecific separation was expected and should not be interpreted as an unexpected biological finding. The observed distribution may reflect the combined effects of taxonomic identity, regional origin, genotype, and environmental conditions, which could not be independently disentangled in the present sampling design.

PLS‐DA and OPLS‐DA were subsequently used to further characterize the discrimination among the sample groups. The PLS‐DA score plot (Figure 2C) showed a grouping pattern broadly consistent with that observed in the unsupervised PCA. The 100‐permutation test was used as an internal diagnostic of PLS‐DA model stability (Figure 2B) (Wheelock and Wheelock 2013). The OPLS‐DA models also showed clear separation among the analyzed groups (Figure 2D; Table 2), with *R* ^2^ *Y* values ranging from 0.996 to 1.000 and *Q* ^2^ values ranging from 0.984 to 0.994. These exceptionally high *Q* ^2^ values indicate strong cross‐validated separation within the present dataset but should be interpreted cautiously. In particular, the large taxonomic and metabolic distance between CFM and the CF groups may contribute substantially to the observed separation, while the limited number of biological replicates may result in optimistic estimates of predictive performance. Although the permutation analysis and the generally consistent grouping observed in PCA provide internal support for the model stability, these procedures cannot completely exclude model overfitting or replace validation using an independent external sample set. Therefore, the high *Q* ^2^ values should be interpreted as evidence of strong separation within the present dataset rather than definitive evidence of external predictive accuracy.

**TABLE 2:** Values of the statistic parameters obtained for different OPLS‐DA models based on LC‐MS data (in positive mode).^a^

Model classes	OPLS‐DA				Number of DM
	Pre	*R* ^2^ *X* (cum, %)	*R* ^2^ *Y* (cum, %)	*Q* ^2^ (cum, %)	
CF2 vs. CF3	2	55.7	100	98.5	188
CF2 vs. CF4	2	59.0	100	99.0	201
CF2 vs. CFM	2	65.3	100	99.4	231
CF2 vs. CF1	2	54.7	100	98.5	185
CF3 vs. CF4	2	54.9	100	98.7	191
CF3 vs. CFM	2	62.5	100	99.3	225
CF3 vs. CF1	2	58.5	100	99.0	199
CF4 vs. CFM	2	63.8	100	99.3	244
CF4 vs. CF1	2	60.7	100	99.1	200
CFM vs. CF1	2	67.1	100	99.4	248
CF1 vs. CF2 vs. CF3 vs. CF4 vs. CFM	2	31.7	99.6	98.4	139

^a^ Pre is the number of principal components; *R* ^2^ *X* (cum), cumulative fraction of *X* variance explained by the model; *R* ^2^ *Y* (cum), cumulative fraction of *Y* variance explained by the model; *Q* ^2^ (cum), cumulative cross‐validated predictive ability; DM, differential metabolite selected using VIP > 1 and *p* < 0.05. Detailed information on the DMs is provided in Table S2.

A total of 65 differential metabolites were identified among the five sample groups, including 19 secondary metabolites comprising 11 flavonoids, three terpenoids, three phenols, one coumarin, and one alkaloid. The numbers of differential metabolites detected in the pairwise comparisons are summarized in Table S2, with the largest number observed in the CFM versus CF1 comparison. These compounds are regarded as candidate discriminatory metabolites rather than validated biomarkers because their predictive performance has not yet been evaluated using an independent sample set.

Importantly, *C. medica* belongs to Rutaceae within Sapindales, whereas *S. edule* belongs to Cucurbitaceae within Cucurbitales. Their marked metabolic divergence is therefore expected and is consistent with the evolutionary and phylogenetic structuring of plant secondary metabolism (Wink 2003; Vieira et al. 2019). Accordingly, the comparison between the two species was not intended as a biologically equivalent or phylogenetically matched contrast. Instead, *S. edule* was included as a targeted authentication comparator because the partially overlapping Chinese vernacular terms “Foshou” and “Foshougua” may contribute to commodity misidentification. Within this authentication‐oriented framework, the interspecific differential metabolites provide candidate chemical features for distinguishing the two commodities, whereas the differences among CF1–CF4 may support regional quality discrimination. This interpretation is consistent with current LC‐MS‐based food‐authentication strategies (Zhong et al. 2022) and metabolomic approaches used to discriminate citrus materials (Song et al. 2025). Nevertheless, the authentication scope of the present study is restricted to the sample groups examined here and requires further validation using independent external samples.

### 3.3 HCA

**FIGURE 3:**
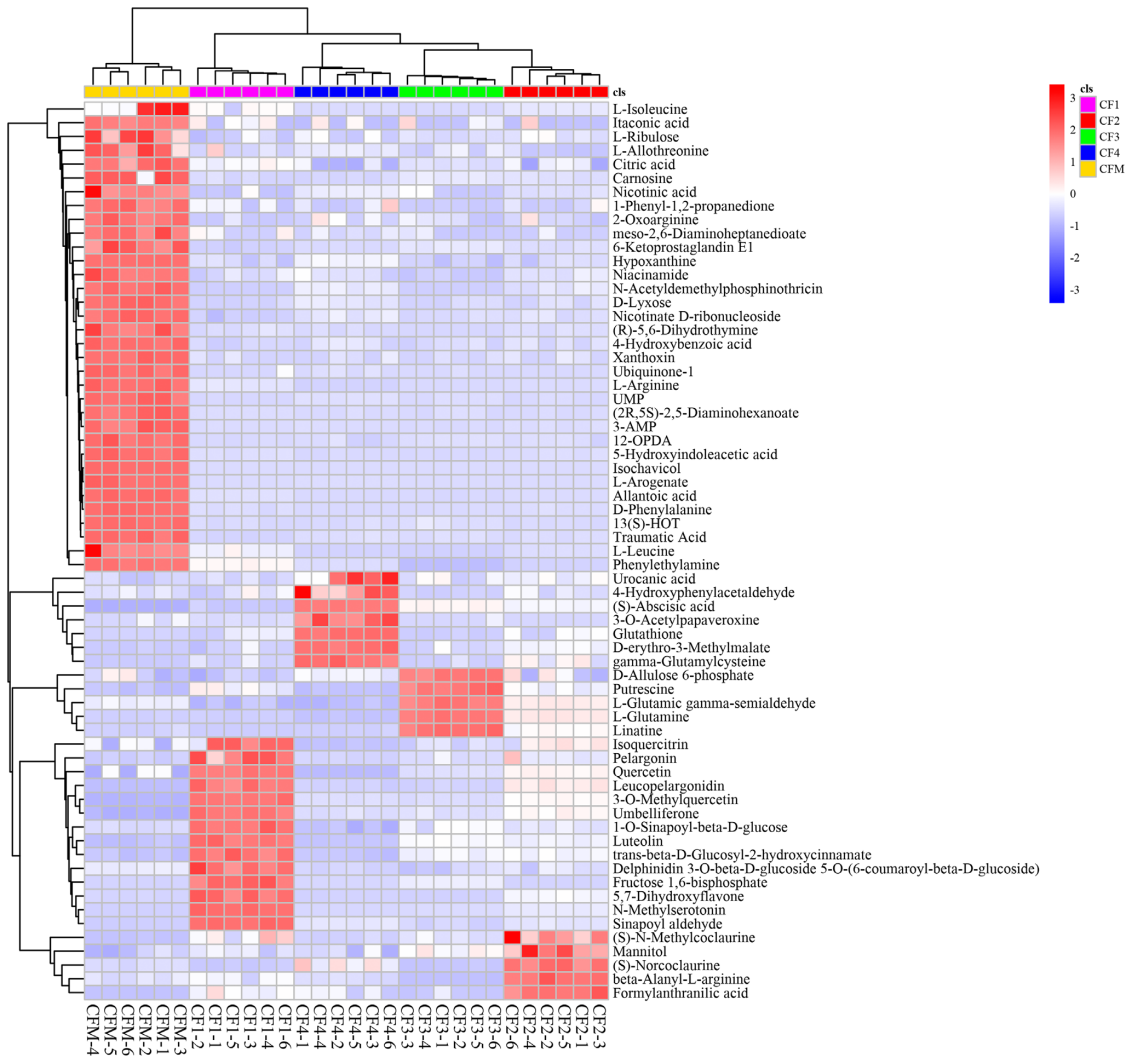
Heatmap of hierarchical clustering analysis of differential metabolites among four regional fingered citron groups (CF1–CF4) and chayote (CFM). The abscissa indicates different groups labeled with different colors for the main groups and numerically marked for the subgroups. The ordinate indicates the differential metabolites selected as candidate discriminatory metabolites in four regional fingered citron groups (CF1–CF4) and chayote (CFM). The bar at the right of the heat map represents row‐scaled relative metabolite abundances.

A total of 65 differential metabolites were used for HCA. As shown in Figure 3, their relative abundance patterns differed among the four regional CF groups and CFM. Thirty‐four of these metabolites showed relatively higher abundance in CFM, whereas the remaining metabolites displayed variable distributions among CF1–CF4. The HCA grouping was generally consistent with the PCA results. Within the present sample set, these 65 differential metabolites represent candidate chemical features for distinguishing CF from the targeted chayote comparator, while the metabolites differing among CF1–CF4 may contribute to regional quality discrimination. However, they should not yet be regarded as validated authentication biomarkers because their sensitivity and specificity have not been evaluated using independent samples.

Since secondary metabolites are important bioactive components of fruits, we further used 19 secondary differential metabolites to conduct the HCA and present the result in Figure S2. Several secondary metabolites, including eight flavonoids, one phenol, one alkaloid, and one coumarin, showed relatively high abundance in CF1. This result suggests that the Zhejiang sample group possesses a distinctive secondary‐metabolite profile. However, metabolite abundance alone is insufficient to establish overall commercial or medicinal quality, and targeted quantification, sensory evaluation, bioactivity assays, and broader multi‐year sampling are required before drawing conclusions regarding regional quality superiority.

### 3.4 KEGG Annotation and Metabolic Pathway Analysis

**FIGURE 4:**
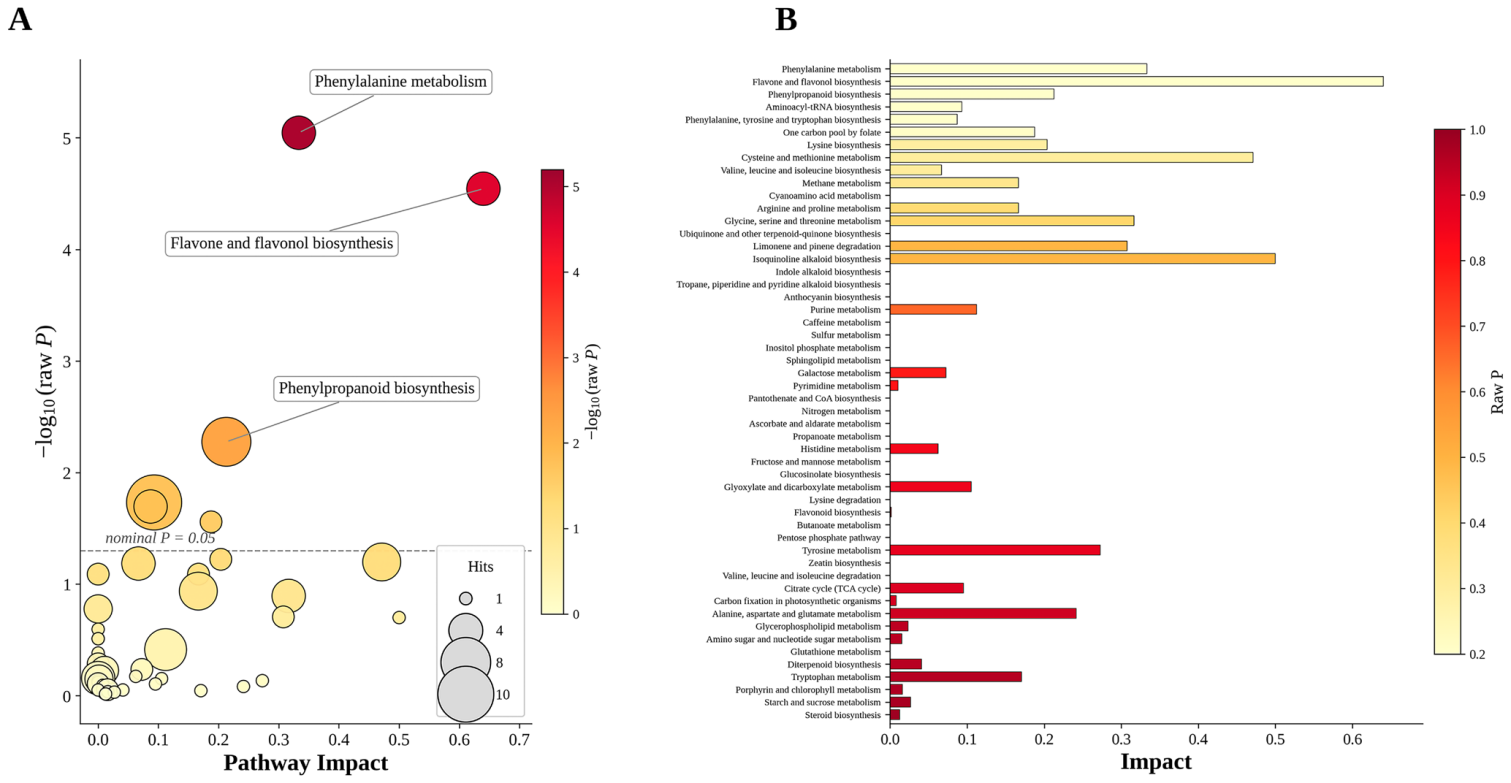
Pathway analysis of differential metabolites in four regional fingered citron groups (CF1–CF4) and chayote (CFM) illustrated as a bubble diagram (A) and histogram (B). In (A), the *y*‐axis and bubble color denote −log10 (raw, nominal *p*), bubble size denotes the number of hits, and pathway impact is shown on the *x*‐axis; the dashed line marks nominal *p* = 0.05. In (B), bar length denotes the pathway impact value obtained from the pathway topology analysis, and bar color denotes the raw (nominal) *p* value of the pathway enrichment test, with paler colors indicating lower *p* values and darker colors indicating higher *p* values (color scale at right); pathways are arranged from top to bottom in order of increasing raw *p* value.

Plant metabolites perform diverse physiological, ecological, and adaptive functions (Luo 2015). Unraveling the metabolic pathways and networks will provide information for the genetic basis of plant metabolism (Carreno‐Quintero et al. 2013). As noted above, both the taxonomic origin (species) and the growing environment influence the accumulation of plant secondary metabolites. In the present study, we compared the metabolite profiles of CF from the different regions and of chayote. The differential metabolites from the comparison were annotated by the Kyoto Encyclopedia of Genes and Genomes (KEGG), a database that integrates genomic, chemical, and systemic functional information (Kanehisa and Goto 2000). The pathway analysis was conducted by MetaboAnalyst (https://www.metaboanalyst.ca/, version 5.0) through integrating pathway enrichment analysis and pathway topology analysis. Figure 4A presents a bubble plot visualizing the pathway analysis of differential metabolites in four regional CF groups (CF1–CF4) and chayote (CFM). The enrichment analysis indicates that pathway of “Flavone and flavonol biosynthesis” has the highest impact (> 0.6) and the *p* value of “Phenylalanine metabolism” pathway is the least (< 0.01). Therefore, although neither pathway remained significant after FDR correction, these two pathways showed the strongest nominal enrichment for the differential metabolites from the comparison of five samples. Figure 4B summarizes all the tested pathways as a horizontal bar chart, in which the bar length represents the pathway impact value obtained from the pathway topology analysis and the bar color represents the corresponding raw (nominal) *p* value; the pathways are ordered from top to bottom by increasing raw *p* value (i.e., decreasing nominal significance).

## 4 Conclusions

In this study, untargeted UPLC‐Q‐Orbitrap MS/MS metabolomics was used to characterize the phytochemical profiles of four regional CF groups and chayote, which was included as a targeted non‐Citrus authentication comparator. A total of 380 metabolites were putatively annotated, including 47 secondary metabolites. Twelve secondary metabolites were tentatively annotated in CF for the first time, to the best of our knowledge. PCA, PLS‐DA, OPLS‐DA, and hierarchical clustering revealed clear differences among the five sample groups and identified 65 differential metabolites, including 19 secondary metabolites. The separation between *C. medica* and *S. edule* was expected because of their substantial taxonomic and metabolic distance. Therefore, this interspecific comparison should be interpreted as a targeted chemical‐authentication analysis rather than a biologically equivalent comparison. The differences among CF1–CF4 additionally provide preliminary information for regional chemical‐quality discrimination. Phenylalanine metabolism and flavone and flavonol biosynthesis showed the strongest enrichment at the nominal *p* value level, although neither pathway remained significant after FDR correction. Consequently, these pathway results should be considered exploratory. Overall, the study provides a metabolite reference for regional CF samples and identifies candidate discriminatory features with potential utility in product authentication and regional quality evaluation. However, the proposed features are not yet validated authentication biomarkers. The study was limited by the inclusion of only one non‐Citrus comparator, a limited number of biological replicates, single‐season sampling, the reliance on database‐level spectral matching without authentic‐standard verification (MSI Level 2), and the absence of independent external validation and direct bioactivity assessment. Future studies should include additional Citrus taxa and potentially confused commodities, multiple harvest years and production sites, targeted quantitative validation, and independent sample sets to determine the specificity, reproducibility, and practical applicability of the candidate markers.

## Author Contributions

**Ruiyi Fan:** investigation, validation, formal analysis, supervision, funding acquisition, writing – review and editing. **Bin Li:** conceptualization, methodology, software, data curation, writing – original draft.

## Funding

This work was financially supported by the PhD Startup Foundation of Jingchu University of Technology (YYZ202512), the Key Project of the Science and Technology Research Program of the Department of Education of Hubei Province (D20244303), and the Jingmen City Innovation and Development Joint Fund of the Hubei Provincial Natural Science Foundation (JCZRLH202601519).

## Conflicts of Interest

The authors declare no conflicts of interest.

## Supporting information


**Figure S1:** Score scatter plots for principal component analysis (PCA) model with all the samples represented by green dots and the quality control (QC) samples represented by red dots in positive mode (A) and negative mode (B). Relative standard deviation (RSD) tests of the detected peaks in positive mode (C) and negative mode (D).
**Figure S2:** Heatmap of hierarchical clustering analysis of 19 secondary differential metabolites. The abscissa indicates different groups labeled with different color for the main groups and numerically marked for the subgroups. The ordinate indicates the differential metabolites selected as candidate discriminatory metabolites in four regional fingered citron groups (CF1–CF4) and chayote (CFM). The bar at the right of the heat map represents relative expression values.

**Table S1:** All compounds identified in this study.

**Table S2:** The information of the differential metabolites compared in various groups (information of each group was provided in individual sheets).

## Data Availability

The data that support the findings of this study are available from the corresponding author upon reasonable request.
