# Supplementary material for: Comparison of the Metabolites in Fingered Citron Fruit (Citrus medica L. var. sarcodactylis Swingle) and Chayote (Sechium edule) Based on UPLC‐Q‐Orbitrap MS/MS
Source: Food Sci Nutr. 2026 Jul 16;14(7):e72115. doi: 10.1002/fsn3.72115 (PMC13376348; doi:10.1002/fsn3.72115)
Supplement: Supplementary file 1 — Figure S1: Score scatter plots for principal component analysis (PCA) model with all the samples represented by green dots and the quality control (QC) samples represented by red dots in positive mode (A) and negative mode (B). Relative standard deviation (RSD) tests of the detected peaks in positive mode (C) and negative mode (D). Figure S2: Heatmap of hierarchical clustering analysis of 19 secondary differential metabolites. The abscissa indicates different groups labeled with different color for the main groups and numerically marked for the subgroups. The ordinate indicates the differential metabolites selected as candidate discriminatory metabolites in four regional fingered citron groups (CF1–CF4) and chayote (CFM). The bar at the right of the heat map represents relative expression values. [file FSN3-14-e72115-s003.docx]

**Supplementary Materials**

**Comparison of the metabolites in fingered citron fruit (*Citrus medica* L. var. *sarcodactylis* Swingle) and chayote (*Sechium edule*) based on UPLC-Q-Orbitrap MS/MS**

Bin Li^1, 2^, Ruiyi Fan^1, 2^ *

^1^ College of Food and Biology, Jingchu University of Technology, 448000, Jingmen, Hubei, China

^2^ Hubei Engineering Research Center for Specialty Flowers Biological Breeding, Jingchu University of Technology, 448000, Jingmen, Hubei, China

College of Food and Biology, Jingchu University of Technology, 448000, Jingmen, Hubei, China; Hubei Engineering Research Center for Specialty Flowers Biological Breeding, Jingchu University of Technology, 448000, Jingmen, Hubei, China

*Corresponding author: fanruiyi@outlook.com (R. F.)

**
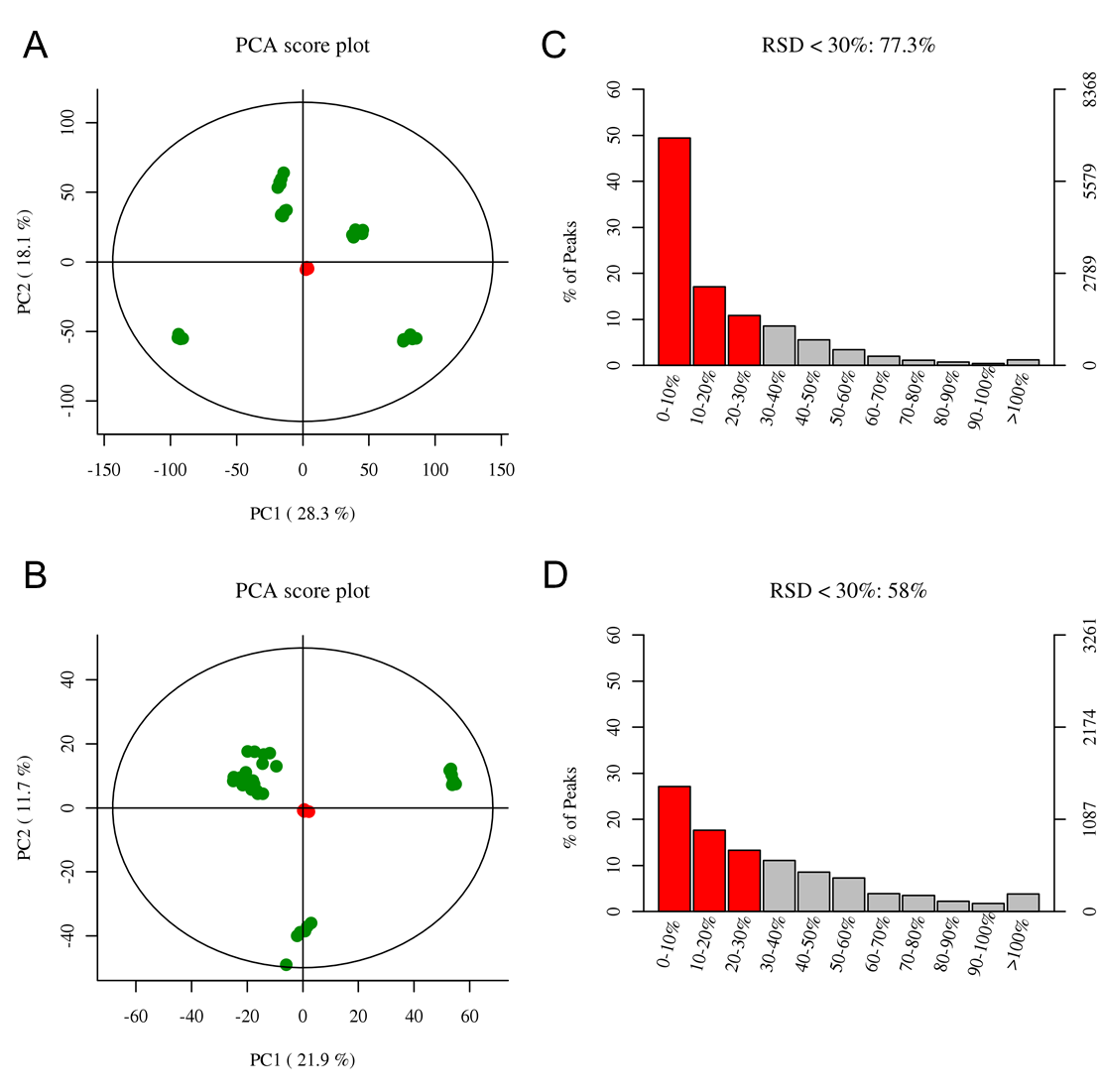
**

**Figure S1** Score scatter plots for principal component analysis (PCA) model with all the samples represented by green dots and the quality control (QC) samples represented by red dots in positive mode (A) and negative mode (B). Relative standard deviation (RSD) tests of the detected peaks in positive mode (C) and negative mode (D).


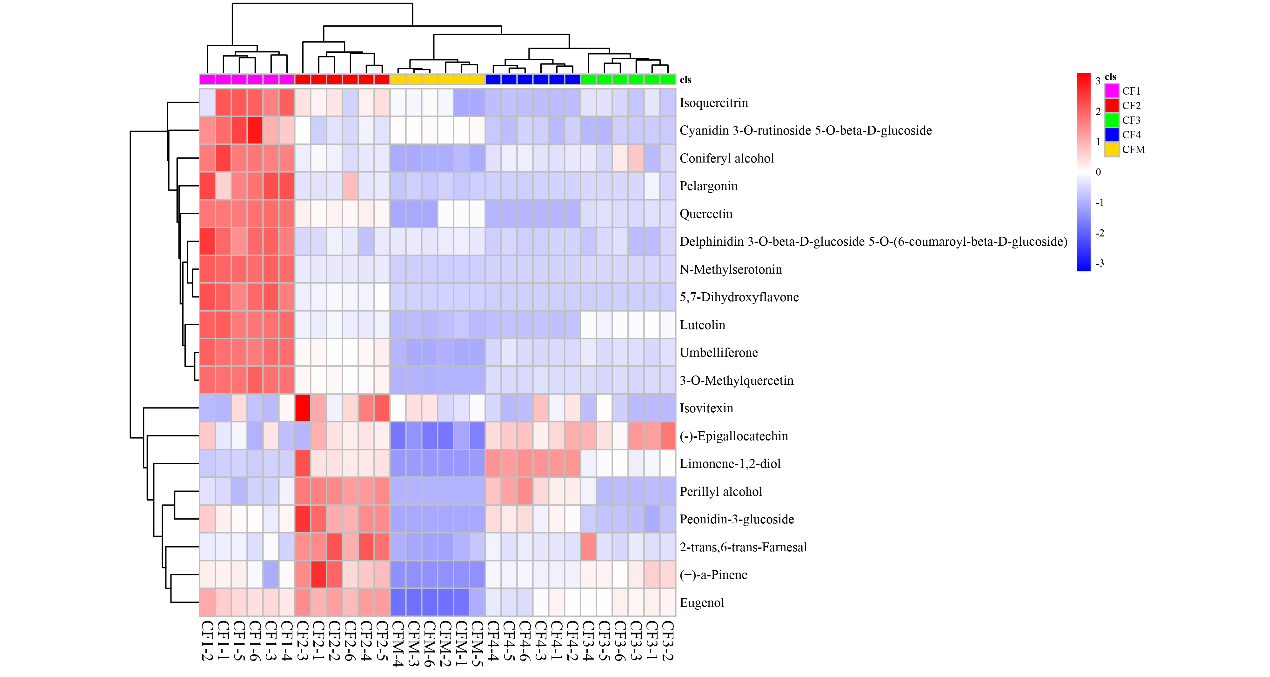


**Figure S2** Heatmap of hierarchical clustering analysis of 19 secondary differential metabolites. The abscissa indicates different groups labeled with different color for the main groups and numerically marked for the subgroups. The ordinate indicates the differential metabolites selected as candidate discriminatory metabolites in four regional fingered citron groups (CF1–CF4) and chayote (CFM). The bar at the right of the heat map represents relative expression values.

**Supplementary Tables caption**

**Table S1.** All compounds identified in this study.

**Table S2.** The information of the differential metabolites compared in various groups (information of each group was provided in individual sheets)
